# Hepatic Iron Quantification on 3 Tesla (3 T) Magnetic Resonance (MR): Technical Challenges and Solutions

**DOI:** 10.1155/2013/628150

**Published:** 2013-05-22

**Authors:** Muhammad Anwar, John Wood, Deepa Manwani, Benjamin Taragin, Suzette O. Oyeku, Qi Peng

**Affiliations:** ^1^Department of Pediatric Radiology, Children's Hospital at Montefiore, Bronx, NY, USA; ^2^Department of Pediatric Radiology, Children's Hospital Los Angeles, Los Angeles, CA, USA; ^3^Division of Pediatric Cardiology, Children's Hospital Los Angeles, Los Angeles, CA, USA; ^4^Department of Pediatrics, Division of Pediatric Hematology/Oncology, Children's Hospital at Montefiore, Bronx, NY, USA; ^5^Department of Pediatrics, Division of General Pediatrics, Children's Hospital at Montefiore, Bronx, NY, USA

## Abstract

MR has become a reliable and noninvasive method of hepatic iron quantification. Currently, most of the hepatic iron quantification is performed on 1.5 T MR, and the biopsy measurements have been paired with *R*_2_ and *R*_2_* values for 1.5 T MR. As the use of 3 T MR scanners is steadily increasing in clinical practice, it has become important to evaluate the practicality of calculating iron burden at 3 T MR. Hepatic iron quantification on 3 T MR requires a better understanding of the process and more stringent technical considerations. The purpose of this work is to focus on the technical challenges in establishing a relationship between *T*_2_* values at 1.5 T MR and 3 T MR for hepatic iron concentration (HIC) and to develop an appropriately optimized MR protocol for the evaluation of *T*_2_* values in the liver at 3 T magnetic field strength. We studied 22 sickle cell patients using multiecho fast gradient-echo sequence (MFGRE) 3 T MR and compared the results with serum ferritin and liver biopsy results. Our study showed that the quantification of hepatic iron on 3 T MRI in sickle cell disease patients correlates well with clinical blood test results and biopsy results. 3 T MR liver iron quantification based on MFGRE can be used for hepatic iron quantification in transfused patients.

## 1. Introduction 

Presence of iron in the body is essential as it forms an important component of metabolic and biological processes. On the contrary, its excess can be a serious health risk for chronically transfused patients, for example, thalassemia and sickle cell disease patients [1, 2]. End organ damage can result from deposition of excessive iron in organs such as hepatic parenchyma, endocrine organs, and cardiac cells [3–5]. Total body iron load is the major determining factor of clinical outcome in all forms of systemic iron overload. Accurate assessment of total body iron load is crucial for managing iron chelation therapy to avoid iron toxicity while preventing the toxicity of excess chelator administration. 

Serum ferritin concentration, transferrin saturation, and serum iron concentration are serum makers for biochemical measurement of total body iron load. In most cases, these correlate well with the total iron burden; however, certain factors like infection, inflammation, and malignancy could modify these, and therefore these may not always correctly reflect tissue iron levels [6, 7]. 

Hepatic iron concentration has been shown to be a reliable pointer of total body iron stores in patients with transfusion-related iron overload. Repeated assessments of the hepatic iron concentration can offer a quantitative means of gauging the long-term body iron balance [8]. Currently, chemical analysis of needle biopsy specimens is the most common accepted method of measurement. However, results may be affected by small sample size by the needle and uneven distribution of iron in the liver [9, 10]. Also, there is a small but finite risk associated with the liver biopsy and lack of patient and parent acceptability [11].

MRI determination of hepatic iron content is a well-validated predictor of iron overload related hepatic complications. The superparamagnetic properties of iron deposited in the liver cause shortening of *T*_2_ relaxation times of the liver, which results in reduction of the signal intensity of the hepatic parenchyma [12]. It has been established that MRI *T*_2_/*R*_2_ and *T*_2_*/*R*_2_* mappings accurately estimate hepatic iron concentration in transfusion-dependent thalassemia and sickle cell disease patients. Currently, most of the hepatic iron quantification is performed on 1.5 T MR, and the biopsy measurements have been paired with *R*_2_ and *R*_2_* values for 1.5 T. Moreover, data related to quantification of hepatic iron on 3 T in sickle cell patients is not yet available. Therefore, the rising use of 3 T MR scanners in clinical practice makes it necessary to optimize and standardize the assessment of iron burden at 3 T.

The goal of this study is to focus on technical challenges in establishing a relationship between *T*_2_* values at 3 T and 1.5 T and hepatic iron burden and to optimize appropriate MR protocol for the evaluation of *T*_2_* values in the liver at 3 T field strength of the magnet.

## 2. Methods

### 2.1. Population

Twenty-two consecutive patients with sickle cell disease (7.6 to 20.9 yrs; 6 females) presenting for MRI assessment of the liver between 4/16/2010 and 8/26/2010 were retrospectively reviewed in this HIPPA compliant study. The study was approved by our institution's review board with a waiver of informed consent. Each patient went through a clinical MRI study to evaluate liver iron load with a multiecho gradient-echo sequence performed as part of the MRI protocol, and correlation was made with serum ferritin and with liver biopsy results when available as a gold standard of the liver iron level.

#### 2.1.1. Measurement of Clinical Parameters

Patients had the MR study done either as part of a pretransplant evaluation or to monitor the efficacy of ongoing chelation therapy. Since analyses were done on a convenience sample, we utilized retrospective, clinically available data to correlate with the report of iron quantification on imaging. We examined transfusional iron load (TIL), liver biopsy results (B) when available, serum ferritin levels (F), and liver function (LFT) abnormalities such elevated alanine aminotransferase (ALT) levels, chelation history (C), and compliance and phlebotomy or exchange transfusions. The number of years of monthly transfusions was recorded for patients on monthly simple transfusions. This data was recorded as not applicable (NA) if chronic transfusion therapy had never been initiated.

### 2.2. Transfusional Iron Load

TIL was calculated based on cumulative blood volume received by patients on simple transfusions. This is one of the benchmarks of iron loading, and one mL of blood of red blood cells is estimated to contain 1 mg of iron [13]. TIL was reported in mL/kg body weight of the patients. It is recommended, and it is our standard practice that a patient that has a TIL > 100 mg/kg be started on chelation therapy [14]. In these patients the total iron content is a balance between ongoing transfusion and chelation therapy, and it cannot be predicted based on TIL alone. Also, patients that are on either exchange transfusions or have regular phlebotomy done will have lower iron burdens than would be predicted from the TIL. We predicted, based on published data [15], that patients with a TLC < 100 mg/kg would have liver iron content in the normal range or <4 mg/g. 

### 2.3. Liver Iron Concentration by Liver Biopsy

The second benchmark of iron overload is the liver iron concentration measured from liver biopsy samples. Liver biopsies (B) were assayed by dynamic reaction cell-inductively coupled plasma mass spectrometry [16]. This information was available on 5 patients. In patients where the biopsy was performed within 6 months, we expected that the 2 measurements would be within 2 mg/g of each other. In the patients where the biopsy was done 6 months or longer from the MRI, we predicted that the biopsy results would predict whether or not the liver iron on *T*_2_* would be less than or 10 mg/g or higher.

### 2.4. Serum Ferritin

Serum ferritin is a widely used and readily available clinical assay used to monitor iron load. It is used as a surrogate marker of intracellular ferritin, a hollow shell protein made of 24 heavy (H) or light (L) subunits that sore iron [17]. Since serum ferritin values can fluctuate, an average of 3 values closest to the time of imaging was used, ignoring any outliers. Analysis of chronically transfused patients on the STOP and STOP2 trials showed that a ferritin level <750 ng/mL was associated with no iron loading, and 750–1500 ng/mL was correlated with low transfusion burden and low measured hepatic iron concentration by biopsy, while a ferritin >3000 ng/mL was consistently predictive of liver iron concentrations of 10 mg/g and a high transfusional burden [15]. In patients with intermediate ferritin values of 1500–3000 ng/mL, true iron content cannot be predicted.

### 2.5. ALT

ALT levels are monitored monthly in patients on chronic transfusions in our practice, and an average of 3 values closest to the MRI study were recorded. Abnormal ALT values was used as a measure of liver injury, which, while not specific, have been associated with iron burden >10 mg/g if obtained at steady state and not during acute illness. In all patients with ALT values that were abnormal, we predicted a hepatic iron burden of >10 mg/g [18].

Compliance with chelation therapy is difficult to measure as there is no serum level of the chelation agent that can be monitored. Lack of compliance was established based on patient report of missing >30% of doses or >30% missed clinic visits.

### 2.6. MR Imaging

All MRI studies were carried out on a single 3.0 Tesla MR unit (Signa HDxt, GE Healthcare) with an 8-channel torso coil. Liver was imaged at three different locations. The first slice was positioned just below the diaphragm through the right lobe of liver, and the next two slices were spaced 8 cms from the first one. A series of images was acquired with increasing echo times (TE) at each slice position using single breath held multiple-echo fast gradient-echo MRI (MFGRE) technique. 

Images were acquired using a single GRE sequence with TR = 30 ms, slice thickness 8 mm, FOV = 400 × 320 mm, and readout bandwidth = 125 kHz. To minimize magnetic susceptibility effect, a small volume shim technique locally over the liver was applied (REF: Storey study) [19].

The examination was monitored for accelerated signal loss in the liver parenchyma. If there was accelerated signal loss in the region of interest (liver), a shorter TE range was used to acquire a repeat acquisition. To achieve this, three matrix sizes 256 × 256, 128 × 128, and 64 × 64 giving an echo spacing of ΔTE *≈* 1.4, 0.7 and 0.5 msec, respectively, were used. Once an acquisition was obtained, the images were visually inspected on the console. In cases where the signal in the region of interest reaches noise level by the second or third echo, a shorter TE range was used to obtain a repeat acquisition. Postprocessing procedure was performed on the GE Report Card 4.0 workstation. A *T*_2_* map was created for each image slice using all 16 TE acquisitions.

### 2.7. MR Image Analysis

*T*_2_* quantifications were performed on the GE Report Card 4.0 workstation. The regions of interest (ROIs) were drawn on first images and were spread automatically through all the images in the corresponding multiecho series. Free hand technique was used to draw region of interest on liver parenchyma. Care was taken to omit vessels, hepatic masses, and artifacts. In addition, peripheral liver parenchyma was not included in the ROIs so as to mitigate the susceptibility artifact arising from air in the lungs. It was also useful to minimize signal variability from adjacent structures.

*T*_2_* values were recorded based on the average numerical values generated from automated curve-fitting for the *T*_2_* decay for each ROI. Three *T*_2_* values were generated from 3 slices for each patient, and a mean *T*_2_* value was obtained by averaging these values. In cases of rapid *T*_2_* signal decay, the data were truncated at the point where the mean intensity fell to the noise level. Finally, the *R*_2_* value for each patient was calculated as the reciprocal of the mean *T*_2_*. 

 We used the following equation to convert the *R*_2_* obtained at 3 T to *R*_2_* of 1.5 T magnet field strength based on the work of Storey et al. [19]:

(1) $$\begin{array}{c} R_{2}^{*}\left(3\;\text{T},C_{\text{Fe}}\right)=2R_{2}^{*}\left(1.5\;\text{T},C_{\text{Fe}}\right)-R_{\text{d-d}}, \end{array}$$

where *R*_d-d_ denotes the relaxation due to diploe-dipole interaction, which was estimated to be 11 ± 4 second^−1^ in the liver. Therefore, the following equation was used to obtain *R*_2_* of liver at 1.5 T:

(2) $$\begin{array}{c} R_{2}^{*}\left(3\;\text{T},C_{\text{Fe}}\right)=\frac{\left(R_{2}^{*}\left(1.5\;\text{T},C_{\text{Fe}}\right)+11\right)}{2.0}. \end{array}$$

The ultimate LIC estimation was obtained using the relationship of 1.5 T *R*_2_* and LIC as described by Wood et al. study [10] with the following equation:

(3) $$\begin{array}{c} \text{LIC}\left(\text{in}\;\text{mg}/\text{g}\right)=0.0254\times R_{2}^{*}\left(1.5\;\text{T}\right)+0.202. \end{array}$$

#### 2.7.1. Statistical Methods

The primary outcome variable was measured liver iron concentration quantified by *T*_2_*. Independent variables include transfusional iron load, liver iron content based on liver biopsy serum ferritin, and presence of abnormal ALT level. Transfusional iron load was divided into two groups: <100 mg/kg and >100 mg/kg. Presence of abnormal ALT level was a binary variable. Ferritin levels were categorized in four groups: <750 ng/mL; 750–1500 ng/mL; 1500–3000 ng/mL; and >3000 ng/mL. 

 Summary statistics were performed to determine means and medians of liver iron content by *T*_2_* and liver biopsy. Frequencies were calculated for key covariates including transfusional iron load, serum ferritin, and ALT. Two-sample *t*-test with unequal variances was used to examine the differences in the mean liver iron concentration quantified by *T*_2_*, tranfusional iron load, and ALT level. Spearman rank correlations were performed to measure associations between liver iron concentration by *T*_2_ and liver iron concentration by liver biopsy greater than 6 months after the MRI. There were insufficient numbers of patients (*N* = 2) who had a liver biopsy within 6 months of the MRI to test an association with liver iron concentration by *T*_2_*. Kruskal-Wallis test was performed to assess whether median liver iron concentration by *T*_2_* differed based on ferritin levels. We performed analyses using STATA/SE 9.2 (StataCorp, College Station, TX, USA). 

## 3. Results

Table 1 lists the clinical characteristics of the 22 patients included in this paper. All reported parameters were described above in the Methods section. The median liver content by *T*_2_* was 3.4 ms, IQR 6.9. (Mean 6.7 ms, SD 6.7).

### 3.1. Association of TIL with MRI Measurement of Hepatic Iron Content

Patients 1 through 9 were not on chronic transfusions, and their TIL < 100 mL/kg. All 9 patients had hepatic iron content on MRI of <4. Patients 11 and 12 were on exchange transfusions and chelation, and one would expect a low iron burden in them as well. Forty-five percent of the study sample (*N* = 10) had a transfusional load greater than 100 mL/kg. The mean liver content by *T*_2_* was significantly higher among patients with a tranfusional load >100 mL/kg (mean 12.3 SD 6.3) compared to patients with a transfusional load <100 mL/kg (mean 2.0 SD 0.86), *P* < 0.0003. 

### 3.2. Liver Biopsy

Patients 10 and 15 had liver biopsies within 6 months of the MRI study. In both, iron quantifications by biopsy and MRI were within 2 mg/g of each other. In the other 3 patients, the iron content on liver biopsy was in the range from 14.2 to 32 mg/g, and the 2 studies were not within 6 months of each other. 2/3 patients had liver iron in the >10 mg/g range as predicted and one had a lower hepatic iron content on MRI. There is a statistically significant relationship between the liver iron content by *T*_2_ and the liver iron content obtained by liver biopsy more than 6 months from the MRI, (*r*s [22 = 0.46, *P* = 0.03).

### 3.3. Ferritin

Among the 22 patients, 32% (*N* = 7) had a ferritin level less than 750 ng/mL, 18% (*N* = 4) had a ferritin level between 750 and 1500 ng/mL, 27% (*N* = 6) had a ferritin level between 1500 and 3000 ng/mL. Twenty-two percent (*N* = 5) had an elevated ferritin of greater than 3000 ng/mL. The median liver content by *T*_2_* significantly differed by ferritin levels (*P* = 0.0004). Patients with ferritin levels greater than >3000 ng/mL had a higher median liver content by *T*_2_*.

### 3.4. Abnormal ALT Levels

Twenty-three percent of patients had an abnormal ALT level (*N* = 5). 

2 of these 4 patients had *T*_2_* liver iron measurements in the predicted range of >10 mg/g. The mean liver content by *T*_2_* was significantly different among patients with an abnormal ALT level (mean 15.9, SD 5.7 compared to patients with a normal ALT level (mean 3.9 SD 4.0), *P* < 0.003. 

### 3.5. Patients with Normal Hepatic Iron Burden on *T*_2_* MRI (<4 mg/g Liver Dry Weight)

Patients 1 through 9 did not have a history of chronic transfusions, and TIL did not exceed 100 mg/kg. Therefore, it is not surprising that none of these patients had the evidence of liver iron loading on *T*_2_* imaging. It is further consistent that the majority of them had ferritin levels less than 750 (patients 1 through 7). 2 patients had intermediate ferritin levels, but none greater than 3000, which is the level more reliably associated with iron overload.

Patients 10, 11, and 12 had no evidence of iron overload despite a history of transfusions and a TIL > 100 mg/kg. Patient 10 had a liver biopsy that closely matched the MRI results. Patient 11 had a low serum ferritin, partial exchange transfusions, and was compliant with chelation therapy. Patient 12 had a short history of transfusions (one year) and had only received exchange transfusions.

### 3.6. Patients with Mild Hepatic Iron Burden on *T*_2_* MRI (4–7 mg/g Liver Dry Weight)

 Patients 13 and 14 had mild overload. This was consistent with their clinical history of discontinued transfusions after one year in patient 11 and ongoing chelation in patient 14.

### 3.7. Patients with Moderate Hepatic Iron Burden on *T*_2_* MRI (7–10 mg/g of Liver Dry Weight)

Patient 15 had a liver biopsy that correlated well with the MRI quantification. Patient 16 had a liver biopsy that reported a higher iron burden. This patient had not been on chronic transfusions but had frequent intermittent transfusions and did not have elevated LFTs, both factors arguing against very severe iron overload. Iron loading in the liver has been documented to be patchy; thus, the area sampled may affect the results. Patient 17 was unable to tolerate chelation secondary to side effects, and it is certainly plausible that based on her ferritin values and TIL, she should have some degree of iron overload. We were able to successfully initiate chelation therapy soon after the MRI imaging. With therapy, she had a decline in her ferritin values, and repeat imaging a year later documented an improvement as well.

### 3.8. Patients with Severe Hepatic Iron Burden on *T*_2_* MRI (>10 mg/g of Liver Dry Weight)

Patient 18 had a long history of noncompliance with chelation therapy. He also had high ferritin levels and intermittently elevated ALT levels. Following the *T*_2_* imaging, he was switched from monthly straight transfusions to scheduled exchange transfusions. 3–6 months after being put on exchange transfusions, his ferritin levels stabilized and slowly started to improve. Also his ALT levels normalized.

Patient 19 had a liver biopsy 1.5 years prior that showed extreme iron overload. The improvement noted on the MRI reflects the change in his treatment plan from transfusions to hydroxyurea (HU) and chelation therapy. The low ferritin levels in his case exemplify the unreliability of the s, ferritin values in the 1500–3000 range. Patient 20 and Patient 21 have extremely high ferritin values, very long history of chronic transfusions and were not on any chelation therapy due to toxicity. His liver biopsy 3 years ago showed extreme iron loading as well. Patient 22 showed extremely high levels of iron on MRI. This extreme iron loading was concordant with her extremely high ferritin levels, abnormal LFTs, and poor compliance with chelation. Also her transferring saturation was 100%, and cardiac *T*_2_* was in the borderline range.

Of the 22 patients studied, only 3 had borderline or abnormal cardiac results, and all 3 patients had severe liver iron loading. The iron loading in the heart in our cohort occurred at a lower rate than in patients with thalassemia major and is consistent with published data [20, 21].

## 4. Discussion

Excessive body iron is a major contributor responsible for morbidity in transfusion-dependent patients. Likewise, excessive chelation has its own toxic effects. Serum makers such as serum ferritin concentrations have poor specificity for assessment of body iron. Biochemical assessment of iron in a liver biopsy specimen is the most reliable method to calculate the body iron. However, the invasive nature of liver biopsy makes it a less desirable procedure, especially for repeated assessment of hepatic iron concentration.

Various imaging techniques have been developed for the detection and quantification of hepatic iron noninvasively. The superconducting quantum interference device (SQUID) very precisely quantify hepatic iron concentration and has shown great correlation with HIC as measured by biopsy [22]. However, this device is expensive and not available for commercial use. *T*_2_ MRI is another technique which has been validated as a reliable noninvasive means to evaluate iron stores in the liver and heart [23]. MR quantification is now considered as the study of choice because of its noninvasive nature.

With the increasing availability of 3 T MR scanners, there is growing need to assess the practicality of evaluating iron burden at 3 T. Although, assessment of cardiac and hepatic iron burden by *T*_2_*/*R*_2_* imaging is feasible at 3 T, yet is less straightforward than at 1.5 T. Therefore there is a need to optimize the MR protocol for the evaluation of *T*_2_* values in the liver and heart at 3 T field strength of the magnet and establish a relationship between *T*_2_* values at 3 T over a range of tissue iron concentrations. We propose using the equation described to convert the 3 T data to LIC values previously determined on 1.5 data based on the work of Storey et al. [19] which had described the relationship between 1.5 and 3 T values of *R*_2_*. 

Our study was limited by several factors. Firstly, a small sample size may have resulted in an unseen bias in the study relating to stage of disease or disease etiology, which would have been reduced by a larger sample size. An additional limitation was a lack of direct correlation of 3 T results with 1.5 T results. Further confirmation may be needed by simultaneous comparison with 1.5 T and 3 T *R*_2_* values.

Lastly, liver biopsy results for the liver iron load were not available for all the patients. However, iron level assessment based on *T*_2_*/*R*_2_* values at 1.5 T has been validated as a reliable method to measure hepatic iron load. Therefore, we felt that the absence of direct correlation with liver biopsy results was not a major limitation. 

In this work, we presented an MRI approach at 3 T to measure *T*_2_* accurately by using different echo time ranges. We also applied a conversion method to estimate hepatic iron concentration based on known *R*_2_* relationship of liver parenchyma at 1.5 T and 3 T. Our study showed that quantification of hepatic iron on 3 T MRI in sickle cell disease patients correlates well with clinical blood test results and biopsy results. We conclude that hepatic iron quantification based on MFGRE at 3 T MRI is feasible in transfused patients. Continued research and multi-institutional validation would be helpful for confirming its validity.

## Figures and Tables

**Table 1:** Summary of imaging and clinical data.

	*T*_2_* at 3 T	*R*_2_* at 3 T	*T*_2_* hepatic LIC in mg/g	Ferritin (mode)	Liver biopsy LIC mg/g dry weight	Documented transfusion iron loading mg/kg	Duration of chronic transfusion in years	ALT	Chelation	Clinical correlation
1	17.4	57.5	1.1	<750		<100	NA	N	NI	F, TIL
2	14.9	67.1	1.2	<750		<100	NA	N	NI	F, TIL
3	14.6	68.3	1.2	<750		<100	NA	N	NI	F, TIL
4	14.7	73.2	1.3	<750		<100	NA	N	NI	F, TIL
5	12.9	77.3	1.3	<750		<100	NA	N	NI	F, TIL
6	11.3	88.2	1.5	<750		<100	NA	N	NI	F, TIL
7	7.4	135.1	2.1	<750		<100	NA	N	NI	F, TIL
8	7.3	137.0	2.1	1500–2250		<100	NA		NI	TIL
9	5.5	183.5	2.7	2250–3000		<100	1	N	NI	TIL, ferritin 1 year. after chronic transfusion were 6000, and followup *T*_2_* was 5 mg/g
10	5.7	176.5	2.6	750–15.00	2.5	>100, switched to HU	1	N	Phlebotomy	B (within 6 months), F
11	4.8	208.3	3	750–1500		>100 (exchange transfusions)	6	N	Y	F, TIL
12	3.7	270.3	3.8	2250–3000		>100 (only exchange transfusions)	2	N	Y	TIL
13	2.9	344.8	4.7	750–1500		>100,	1		N	F, received 12 monthly transfusions for one year, discontinued a year prior
14	2.5	400.0	5.4	2250–3000		>100,	4	N	Y	
15	1.7	600.0	8	2250–3000	6.3	>100,	2	N	N	B (within 6 months),
16	1.6	625.0	8.3	2250–3000	14.2	>100,	4	N	Y,	F, and ALT. Poor compliance
17	1.57	635.0	8.4	>3000		>100,	5	Y	N	F, repeat *T*_2_* a year after chelation 6.9
18	1.2	833.3	10.9	>3000		>100,	9	Y	Y	F, ALT LFTs improved after he started on exchange, poor compliance
19	0.8	1304.3	16.9	2250–3000	24	>100, switched to HU	11	N	Phlebotomy	B done 1.5 years prior, after which patient switched to HU
20			Extreme iron overload	6–8000		>100,	15	Y	Y	F, ALT, and poor compliance
21			Extreme iron overload	>10.000	32	>100,	16	Y	Contraindicated due to toxicity	B (3 years prior), F,
22			Extreme iron overload	8–1100		>100,	8	Y	Y	F, ALT Poor compliance

F: ferritin, TIL: transfusional iron loading, abnormal LFTs: ALT, B: liver biopsy results, and NI: not indicated.

## Abbreviations

HIC: Hepatic iron concentration
F: Ferritin
TIL: Transfusional iron loading
C: Chelation status
LFTs: Liver function tests
ALT: Alanine transaminase
B: Liver biopsy results
MRI: Magnetic resonance imaging
MFGRE: Multiecho fast gradient-echo sequence
FOV: Field of view.
