# Corrigendum to “Hepatic Iron Quantification on 3 Tesla (3 T) Magnetic Resonance (MR): Technical Challenges and Solutions”

**DOI:** 10.1155/2018/8142478

**Published:** 2018-06-03

**Authors:** Muhammad Anwar, John Wood, Deepa Manwani, Benjamin Taragin, Suzette O. Oyeku, Qi Peng

**Affiliations:** ^1^Department of Pediatric Radiology, Children's Hospital at Montefiore, Bronx, NY, USA; ^2^Department of Pediatric Radiology, Children's Hospital Los Angeles, Los Angeles, CA, USA; ^3^Division of Pediatric Cardiology, Children's Hospital Los Angeles, Los Angeles, CA, USA; ^4^Department of Pediatrics, Division of Pediatric Hematology/Oncology, Children's Hospital at Montefiore, Bronx, NY, USA; ^5^Department of Pediatrics, Division of General Pediatrics, Children's Hospital at Montefiore, Bronx, NY, USA

In the article titled “Hepatic Iron Quantification on 3 Tesla (3 T) Magnetic Resonance (MR): Technical Challenges and Solutions” [1], there was an error in equation (2), where the *R*_2_^*∗*^ values for 1.5 T and 3 T are reversed. The corrected equation is as follows:

(2) $$\begin{array}{c} R_{2}^{*}\left(\;1.5\;T,C_{Fe}\;\right)=\frac{\left(R_{2}^{*}\left(3\;T,C_{Fe}\right)+11\right)}{2.0}. \end{array}$$
